# Supplementary material for: Longest sediment flows yet measured show how major rivers connect efficiently to deep sea
Source: Nat Commun. 2022 Jul 20;13:4193. doi: 10.1038/s41467-022-31689-3 (PMC9297676; doi:10.1038/s41467-022-31689-3)
Supplement: Supplementary file 1 — Supplementary Information [file 41467_2022_31689_MOESM1_ESM.pdf]

SUPPLEMENTARY INFORMATION

SUPPLEMENTARY DISCUSSION

After 18 years without a major flood or cable-breaking flow, a series of four cable-breaking flows occurred on January 14-16th 2020, March 9th 2020, April 28-29th 2021, and January 28th 2022 (Figs. 3 and 4; details in Supplementary Table 1). The cable-breaking flows in 2020 and 2021 occurred after major floods in December 2019 (1-in-50 year flood) and December 2020 (1-in-20 year flood)[43], whilst the January 2022 flow occurred after a much smaller peak annual discharge (Fig. 3). In each event, these flow occurred weeks to months after the flood peak. Three cable-breaking flows in 2020-21 are associated with spring tides, especially those in March 2020 and April 2021, which have longer (months not weeks) delays after major flood peaks (Fig. 4). However, the January 2022 flow is not associated with a spring tide (Fig. 4).

Triggers of smaller (non-cable breaking) turbidity currents in upper Congo Canyon

It is unclear whether the slower and shorter runout (non-cable-breaking) flows recorded in the ADCP data are also associated with spring-neap tidal cycles (Supplementary Fig. 3). The timings of turbidity currents were detected first by a mooring located ~80 km from the river mouth (Fig. 1). Transit speeds between first and second moorings were used to infer flow speeds, and hence the time at which flows began at the river mouth (see Methods). For fast moving flows (2-8 m/s), such as the three cable breaking flows, uncertainties in when flows initiated at the river mouth are just a few hours, so correlations to spring-neap cycles are clear. However, for slower moving flows (especially those travelling at < ~0.5 m/s) there may be uncertainties of several days for when flows initiated, complicating correlations to spring-neap tidal cycles. However, for the five fastest (>2 m/s) non-canyon-flushing flows that infilled in the upper canyon, there is a correlation between event timings and greater tidal range (Fig. 4c).

Earlier (1883-1937) cable breaks and turbidity currents in Congo Canyon

Early telegraphic communications cables (1883 to 1937) that crossed the Congo Submarine Canyon followed three routes across the upper canyon, at water depths of <2 km (Supplementary Figure 2a)[71]. Faults on these early cable routes were frequent, and also record turbidity currents in the upper canyon (Supplementary Fig.

2b). The faults were most common during months in which the Congo River discharge was high (Supplementary Fig. 2c)[71]. Thus, there is a weak relationship between months with elevated river discharge and more frequent turbidity currents, which were powerful enough to break those older cables[71]. This again suggests that elevated sediment supply to the estuary plays a role in triggering turbidity currents, although some events occurred long after the flood peak in December. However, this is a broad relationship, and some turbidity currents occur during months of relatively low river discharge, as again seen in 2019-2020 (Fig. 3). Clusters of cable-breaking turbidity currents tended to again occur after one of more years with elevated Congo River discharge (Supplementary Fig. 2d). However, no statistically significant correlation was observed between the timing of these 1883-1937 cable breaks and spring tides at Soyo.

It is also possible to compare the timing of these older cable breaks in the uppermost canyon and information on maximum annual river discharge (Fig. 3a; Supplementary Fig. 2). Cable breaks seem to occur in clusters. On at least one occasion (1925-1931), a series of cable breaks (in 1928 and 1931) continue after three preceding years of high river discharge (1925, 1926 and 1927). This may resemble the pattern of cable breaks both during years of major floods in 2019 and 2020, and after a year of moderate peak river discharge in 2021.

Waves unlikely to trigger large (cable-breaking) turbidity currents in Congo Canyon

Large waves are thought to be able to trigger turbidity currents in submarine canyons[72]. Ocean buoys can provide an excellent record of these phenomena. However, their global coverage is spatially and temporally variable and their data are not always freely available. For the Congo Canyon, we therefore used global model reanalysis data. We used ERA5 hourly reanalysis data of significant wave height (m), mean wave period (seconds), and mean wave direction (degrees) provided by the European Centre for Medium-Range Weather Forecasts (ECMWF) at the head of the Congo Canyon. These data were gridded at 0.5° x 0.5°. These data revealed no clear relationship between significant wave and swell height, mean wave period, or mean wave direction and turbidity current occurrence. It is therefore unlikely that the observed turbidity currents were triggered as a consequence of surface waves or their influence on the seafloor. Indeed, a spit at the entrance

to the estuary tends to protect the head of the canyon (Fig. 1d) from the prevailing wave direction thus reducing the influence of wave triggering at the head of the canyon inside the river mouth/estuary.

Triggering of large (cable-breaking) turbidity currents by river floods and spring tides

This section provides a more complete discussion of how unusually powerful and long runout turbidity currents might be preconditioned by floods of the Congo River, some of which are finally triggered during spring tides (Figs 3 and 4). Four different process-models are outlined for how the offshore turbidity currents could be triggered (Supplementary Fig. 4). It is shown that the first three models are all plausible (Supplementary Fig. 4b-d), but that the fourth model is highly unlikely (Supplementary Fig. 4e). Detailed field observations during 2020-2022 are lacking from the estuarine river mouth, such the exact processes that produced these cable-breaking flows is uncertain. Further field work within the river estuary may be necessary to distinguish between the competing models that are outlined below.

Field observations from times without major floods provide some important insights into sediment transfer from the Congo River to the submarine canyon head (Supplementary Figs. 4a and 5). For example, transfer of sand and mud from the river to the canyon head is decoupled. Sand is supplied mainly as bedload, with the rate of bedload transport into the main canyon head dependent on river discharge, but also increasing markedly during stronger ebb tides [54]. In contrast, finer muddy sediment is carried primarily within a surface plume of fresher water originating from the river, which extends much more widely across the estuarine river mouth[55] (Supplementary Fig. 4a). Most authors have assumed that muddy sediment dominates the annual sediment flux from the Congo River (29-43 Mt/yr[6,44,68]). However, some studies suggest annual rates of bedload transport may be up to 130 Mt/yr near the river mouth[54].

Detailed field observations have also been made for an extensive (~30 km^3) shallow-water (< 12 m) plateau, which is located just upstream from the port of Soyo (Y in Supplementary Fig. 4a) (Nunny *pers. Comm.*, 2021). The muddy surface river plume normally flows above this plateau (Supplementary Fig. 5a). These observations indicate that mud settling occurs across this shallow plateau from the surface river plume. During periods of higher river discharge, the freshwater plume can touch-down on the seabed across the plateau (as increased freshwater discharge displaced saline water seaward), causing remobilisation of the

bed (Supplementary Fig. 5b). When there is a combination of peak river discharge and low tides, this process has been observed to form seaward-flowing fluid-mud layers[56,57] across the plateau. The fluid-mud layers can be several meters thick, and they flow into an adjacent tributary canyon head (Z in Supplementary Fig. 4a).

Four different general models for generation of the large cable breaking turbidity currents are now presented and evaluated, in the light of available field evidence outlined above (Supplementary Fig. 4).

Model 1: Delayed failure of the canyon-lip: The first model is that floods of the Congo River drive large amounts of sand-dominated bedload across the lip of the submarine canyon (Supplementary Fig. 4b)[54]. Rapid and sustained deposition of sand at the canyon-lip would indeed cause it to rapidly build out. The rapidly deposited sediment could then fail as a submarine landslide, to generate a major turbidity current. It is possible that the volume of sediment that fails in such a post-flood landslide is unusually large, and thus produces a particularly fast-moving turbidity current in the canyon head. However, a significant time delay (2 weeks to 4 months) occurs between Congo River flood peaks and all four cable-breaking turbidity currents (Figs. 3 and 4). Thus, although rapidly deposited flood-sediment may prime the submarine canyon-head for failure, it must then remain close to failure for weeks to months, possibly years, until a minor perturbation associated with spring tides triggers final failure[30,49]. Those perturbations might be due to expansion of gas bubbles in sediment[53], although the maximum tidal range of the Congo Estuary (~1.2 m) is not extreme. Alternatively, somewhat increased bedload transport may occur across the canyon-lip at a spring ebb tide[54], potentially causing final failure. This type of model can generate turbidity currents at smaller fjord-deltas, where turbidity currents tend to occur at low tides when river discharges are high[30,48-50]. In these studies of fjords, the timing of turbidity currents was delayed by only by a few hours after the river flood peak[49]. However, studies elsewhere suggest that sediment in submarine canyon heads may remain close to failure for longer periods[72].

Model 2: Mud settling from the surface plume, and remobilisation at later spring tide

A second model is that river floods provide exceptionally large amounts of fine-grained mud, which is then stored within the river-mouth estuary for weeks to months, before being released at spring tides (Supplementary Figs 4c). This mud is initially dispersed via surface plumes[55] (Fig. 1d), and settles onto the

seabed across the entire estuary. Field observations from the shallow water plateau upstream of Soyo (Supplementary Fig. 4a) show that a mud layer accumulates throughout the year (Supplementary Fig. 5). During periods of elevated river discharge, and especially when spring ebb tides also occur, the freshwater plume may touch-down on the shallow water plateau. This causes the previous deposited mud to be resuspended (Supplementary Figure 5b), forming highly-mobile fluid-mud layers[57], which are observed to be several meters thick across the shallow-water plateau near Soyo (Supplementary Fig. 5). These fluid-mud layers then drain into tributary canyon-heads, where they may either directly generate smaller turbidity currents, or produce unstable deposits that episodically fail to produce even larger turbidity currents (Supplementary Figs. 4 and 5).

Model 3: Sediment trapping by near-bed circulation into an estuarine turbidity maximum

A third model, which may act in conjunction with the second model, is that near-bed estuarine circulation[57] traps large volumes of muddy sediment from the river flood, forming an estuarine turbidity maximum (Supplementary Fig. 4d). The near-bed flow direction of water within estuaries converges at the boundary between the outward-directed river plume, and the inward-directed return flow of seawater[57] (Supplementary Fig. 4d), and this flow convergence may act to trap fine near-bed sediment. Work in other estuaries shows how flood sediment may indeed be trapped efficiently by estuarine circulation for at least several weeks after floods[57,73].

This trapped sediment would need to be released mainly at spring tides to explain the timing of the cable breaking turbidity currents in the Congo Canyon (Fig. 4d), which occurred weeks to months after the flood. In other locations, notably the continental shelf offshore from the Amazon River[74], flow convergence can generate thick and highly mobile fluid mud layers[57]. However, these fluid mud layers occur preferentially at neap tides, when such circulation is weaker and there is less turbulent mixing, which allows greater mud settling and stratification[57,74]. Reduced turbulence and mixing at neap tides cannot explain turbidity currents at spring tides in the Congo Estuary (Fig. 4d), and another mechanism is needed to these trigger turbidity currents.

Model 4: Direct plunging of sediment laden (hyperpycnal) river plumes:

Past work on how rivers impact the deep-sea has also focussed on a final model, in which river floodwater contains enough sediment to be denser than seawater, such that it plunges to travel along the seabed (Supplementary Figure 4e)[27]. The Congo River typically has unusually low suspended sediment concentrations for large rivers (e.g. 26-38 mg/l)[52], which are lower than the sediment concentrations needed for the river water to exceed the density of seawater, even via convective fingering (~1,000 mg/l)[27,75]. Thus, this model seems unlikely, although it is possible that suspended sediment concentrations in the river plume may increase somewhat during major flood events.

Triggering of turbidity currents by river floods and spring tides at other locations

The previous section summarises how river floods and tides combine to generate turbidity currents in the Congo River mouth. However, recent work shows that floods and tides are responsible for triggering turbidity currents at a range of other locations[30,48-50], suggesting it is an important and widespread phenomena.

For example, monitoring a set of river-deltas in Canada provides also provides insights into how a combination of elevated river discharge and spring tides may initiate turbidity currents[30, 48-50]. These studies document that turbidity currents are triggered in two different ways. First, floods may lead to increased bedload transport that causes the delta-lip to prograde rapidly and fail[30,48-50], although failure occurs a few hours after flood peaks[49], in a broadly similar way to Model 1 albeit with shorter delays after the flood peak. Second, elevated river discharge and low (ebb) tides produce much stronger offshore-direct river plumes, that carry greater amounts of suspended sediment, which then settles onto the seabed (as in Models 2 and 3)[30,48-50]. Direct monitoring shows how the flux of sediment settling is greatest at slack low tides[30,48-50], as turbulence and mixing in the overlying river plume declines. This settling sediment thus accumulates most rapidly on the seabed at low spring tides, when it may either produce a fluid mud layer that moves downslope, or triggers failure of the seabed, with the exact mechanism being uncertain[30,48-50]. Estuarine circulation may also act to trap finer near-bed sediment, and this process thus resembles Models 2 and 3 outlined above. This is the main mechanism that generates turbidity currents offshore from the Fraser and Squamish Rivers in Canada[30,48-50], where such flows generated via settling from surface plumes

can be more powerful and travel further than turbidity currents generated by delta-lip progradation and failure[76]. This work indicates that turbidity currents can be generated by a wide range of rivers with low (> 70 mg/l) suspended sediment concentrations[48,75].

These field observations at sites in Canada show how turbidity currents typically occur during periods of elevated river discharge, as well as low tides[48-50]. However, powerful turbidity currents in the Congo Canyon can also occur when the river discharge was relatively low, such as on March 9th 2020 and April 28-29th 2021, where they coincide with exceptional spring tides (Figs 4 and 5). It may be that the much larger Congo River is still sufficient to generate turbidity currents at lower discharges, especially if a spring tide is especially low, or if strong sediment trapping by estuarine circulation occurs long after a flood in Model 3.

Previous work suggesting that river floods can trigger turbidity currents that reach the deep-sea

Here we use direct and unusually detailed flow monitoring to document how river floods generate turbidity currents that reach the deep-sea. It is important to acknowledge that previous studies have proposed that floods can produce turbidity currents that reach the deep-sea[27,51,77-78]. However, it is also important to note that this past work was typically based on indirect evidence, and assumed that the deep-sea turbidity currents were triggered by plunging (hyperpycnal) river discharge (i.e. Model 4). This section thus initially outlines why this indirect evidence had considerable uncertainties. It briefly notes turbidity currents in the Congo Canyon occurred after flood peaks, and were not generated by plunging (hyperpycnal) river discharges. The only previous direct evidence that river floods could generate turbidity currents that reached the deep-sea came from cable-breaks in the Gaoping Canyon offshore Taiwan[24,25], although this information is much less detailed than that presented here for the Congo Canyon. The final part of this section thus outlines key insights from offshore Taiwan, and compares them to the results from this study of the Congo Canyon.

Indirect evidence: It has previously been inferred that distinctive features of deep-sea turbidity current deposits (turbidites) record triggering via hyperpycnal river floods[27,77]. These features include patterns of inverse-to-normal grading inferred to record a waxing and waning flood hydrograph[27,77], or shorter-term pulsing of the flow[78]. However, as documented by recent direct flow monitoring, similar patterns of flow

velocity can occur in turbidity currents triggered by both river floods or other processes (e.g. landslides)[79]. Moreover, faster parts of the flow may catch up with slower parts, reorganising and shredding a velocity signal from the initial trigger[79]. For example, Heerema et al.[79] show how turbidity currents associated with Var River floods, or triggered without any river flood (probably by landslides), can have a similar velocity signal. Moreover, pulses within these flows amalgamated within just 16 km of the Var River mouth[79], a much shorter distance than to the first mooring in this study of the Congo Canyon. Thus, as noted by some past authors[51], it may be challenging to infer a river flood trigger from deep-sea turbidite deposits alone.

It has also been proposed that relatively straight submarine canyons and channels may be used to infer turbidity currents triggered by hyperpycnal floods[51]. However, relatively straight canyons or channels may result from other processes. For example, time lapse mapping and flow monitoring in Bute Inlet in Canada shows how relatively straight channels may result from rapid upslope migration of knickpoints[58,63] (water-fall like steps), triggered by turbidity currents unrelated to floods[63,64,80]. So this second line of geomorphic evidence for flood triggering is also uncertain. However, this study strongly supports the view of Piper and Normark[51] that the vast majority of sediment within canyon-flushing flows is derived from seabed (conduit) erosion, rather than originating from within the initial river flood itself (Table 1; Supplementary Table 3).

Direct evidence from cable breaks offshore Taiwan: The only other direct evidence that river floods can generate powerful turbidity currents that reach the deep-sea comes from cable breaks in Gaoping Canyon offshore Taiwan[24,25]. In both 2009 and 2015, multiple cable breaks record turbidity currents that ran out for several hundred kilometres, at speeds of 5-8 m/s (Fig. 6c), to water depths of at least ~4,000 m[24,25]. In both cases, the cable-breaking flows were associated with typhoons and river floods, involving Typhoon Morakot in 2009 and Typhoon Soudelour in 2015[24,25] (Supplementary Fig. 6).

Typhoon Morakot in 2009 produced world-record breaking rainfall, and numerous landslides, causing an exceptional flood along the Gaoping River that transported 280-570 Mt of suspended sediment to the canyon head (Supplementary Fig. 6)[81,82]. This compares to this river's average annual suspended sediment load of just 35-49 Mt[81-83]. Initial cable breaks coincided with the flood peak, and may have resulted directly from plunging hyperpycnal river discharge[24]. However, a second turbidity current with much longer runout

then occurred three days after the Morakot flood peak, when the river was no longer hyperpycnal[24]. Thus, a significant delay occurred between the flood peak and main offshore flow, suggesting the river mouth remained primed by rapid deposition of flood sediment[24]. However, this delay of three days was much shorter than that (three weeks to 4.5 months) in the Congo Canyon system in 2020 and 2021 (Fig. 4d).

A second set of cable breaks in 2015 recorded a turbidity current associated with Typhoon Soudelour[25]. This was a much smaller river flood than in 2009, and it transported a far lower amount of suspended sediment, with sediment and water discharges similar to an average year[81,82] (Supplementary Fig. 6). Unlike the 2009 event, this 2015 turbidity current coincided with the flood peak[25].

Analysis of tidal records, shows cable breaking turbidity currents originating from the mouth of the Gaoping River are not related to spring tides, and occurred after typhoon-driven wave heights had declined[24]. The final trigger for the seabed failure that caused this event in 2009 is thus unclear. This wider comparison between Congo and Gaoping Canyons is instructive, as it suggests that once a river mouth is primed by rapid deposition of flood sediment, a range of different final triggers may then initiate powerful and long runout turbidity currents. The relatively short delay between Morakot flood peak in 2009 and powerful offshore flow may be due to the exceptional amount of sediment supplied in this event to the canyon head (Supplementary Fig. 6). However, smaller river floods can also initiate powerful offshore turbidity currents. These flows from smaller floods may either coincide with the flood peak (as in Typhoon Soudelour in 2015), or occur several weeks after the flood peak (as in Congo Canyon in January 2022).

Detailed bathymetric survey data are not yet available for the Gaoping Canyon, and previous mooring based observations (although ground breaking) were been restricted to the uppermost canyon[84]. Thus, it is unclear how much sediment was carried by these flood-related turbidity currents, and flushed into the deep (> 2 km) sea, or whether changes in flow front speed (Fig. 7c) are driven mainly by variations in canyon width, as can occur elsewhere[35]. Some variations in flow front speeds (Fig. 7c) may be due to cables that broke sometime after flow arrival, as flow speeds cannot be compared to precise ADCP-mooring data. Long runout turbidity currents that break cables in Gaoping Canyon are also triggered by earthquakes (Fig.

7c), unlike the Congo Canyon. These earthquake triggered flows may remove sediment accumulations at the river mouth, which may then affect how much sediment is available for subsequent flood-related flows.

Sediment grain size distributions in Congo Canyon-channel and other systems

Previous work suggested that sediment size (and thus settling velocity) is a primary control on whether turbidity currents ignite, autosuspend or dissipate[36]; and thus runout distances and how efficiently flows transfer sediment to the deep-sea. Therefore, this section now summarises grain sizes in the four field sites in which patterns of front speed have been compared by this study (Fig. 7; also see Supplementary Table 3).

No system is characterised by an individual grain size; instead systems are characterised by a distribution of multiple grain sizes. Indeed, there is a distribution of grains sizes supplied to the system, carried within flows, and deposited on the seabed. Moreover, grain size distributions within a turbidity current will also vary with height above the bed, and with distance from front to back of the flow, further complicating definition of 'representative' grain sizes. Thus, it can be unclear which parts of a grain size distribution, and from which part of a turbidity current, should be used to test past ignition theory[35,36]. A second general issue is the availability of suitable field data for grain size, noting that grain sizes vary spatially across the field site, and indeed within individual cores or sediment trap samples from a single location. For these reasons, this section only summarises approximate fractions of sand (> 63μm) and mud (< 63μm) present at the four field sites (Supplementary Table 3). With this definition it is then possible to determine which systems carry significantly more sand or mud than others, even though it is currently not possible to define exact grain size ranges carried within specific parts of turbidity currents.

Congo Canyon-Channel (~80% mud and ~20% sand): This system is fed by the Congo River, which supplies ~29-43 Mt/yr [6,44,55] of muddy sediment that forms a surface plume extending across the river estuary (Fig. 1d) with a modal (d_{50}) size of 10-20 μm[55]. Coarser sand is also transported as bedload by the river in the canyon head, with a modal size of 200-600 μm[54]. It is assumed commonly that in large rivers such as this, bedload flux is <10% of that carried as muddy suspended load[6,68]. However, Peters et al.[54] inferred that the lowermost Congo River transports up to ~130 Mt/yr of sandy bedload, based on field surveys and samples. Thus, sand could potentially comprise 75% of the Congo River input, if this study is correct[54]. More

generally, this large discrepancy shows how sediment fluxes in large rivers can be highly uncertain, and lead to similar uncertainties in estimates of global annual sediment flux from rivers to the ocean (Table 1; [6,68]). However, observations of thick fluid mud layers offshore Soyo show that the Congo system receives far greater amounts of mud than Monterey Canyon or Bute Inlet, where such thick fluid mud layers are lacking[34,35,85-89]. It is also clear from sediment cores that the floor of the upper Congo Canyon is dominated (> 80%) by mud[32], and it is far muddier than the floor of Monterey Canyon or Bute Channel (both > 90% sand)[80,85,89]. Deeper-water parts of the Congo Channel contain more sand[86], but sediment reaching the terminal lobe comprises 87% mud and 13% sand[39]. Sediment trap samples that recovered sediment from ~35 m above the bed in smaller-scale (Oct-Dec 2019) flows, are also dominated (> 90%) by mud with modal grain sizes of ~10-30 μm (M. Baker, *pers. comm*, 2022). It is thus estimated that turbidity currents in the Congo Canyon comprise approximately 80% mud and 20% sand, albeit with uncertainties, and noting much high sand fraction might occur in dense near-bed layers.

Monterey Canyon (~10% mud and ~90% sand): Monterey Canyon is supplied primarily (~85%) by longshore drift that drives ~1.2 Mt of sand into the canyon head[34,72]. This system therefore contains a significantly larger fraction of sand than the other three river-fed systems. This is consistent with sediment cores from the canyon axis that are dominated by sand and coarser sediment (d_{50} of 200-600 μm) from water depths of 100 m to >1850 m[34,85]. Sediment traps that captured sediment carried within the lower ~15 m of flows contain thick layers of coarse sand[85], suggesting that these turbidity currents carried a higher sand fraction than those in the upper Congo Canyon. However, traps also show that significant amounts of mud (d_{50} 10-25 μm) are resuspended by internal tides within Monterey Canyon between turbidity current events[85]. This mud is not well represented in canyon floor deposits and cores, which lack the meter-thick fluid mud layers seen in Congo Canyon and river-mouth. Deposits at progressively greater heights above the floor of Monterey Canyon become dominated by mud[85,87]. Thus, mud is transported in turbidity currents moving down Monterey Canyon, but these flows are likely dominated by sand (especially near the bed). The sand-dominated floor of Monterey Canyon may also have notably different geotechnical properties than the mud-dominated floor of the upper Congo Canyon, which may affect processes of seabed erosion[35].

Gaoping Canyon (~50% mud, ~50% sand): Sediment supply is dominated by the Gaoping River, whose mouth is immediately adjacent to the canyon-head[84]. The river has a mountainous catchment, and during extreme floods it has sufficiently high sediment concentrations to form plunging (hyperpycnal) offshore flows[24,84]. On average the river supplies ~40 Mt of suspended sediment each year, which is mainly mud (< 63 µm) with subordinate fine sand[84]. However, some typhoons generate exceptionally large flood discharges, which carry far more sediment. For example, it is estimated that Typhoon Morakot in 2009 produced an extreme flood that carried 280-570 Mt of suspended sediment to the canyon head, over just a few days[81,82]. This is around ten times the average annual sediment load. During such exceptional floods, the modal size of suspended sediment may increase to be fine sand, albeit still with a large mud fraction[81-82,84]. There is little information on bedload transport rates, especially in major floods, although bedload may be ~30% of the suspended load[83]. The bed of the river comprises coarse sand near its mouth[84]. Sediment cores from the floor of the upper canyon contain thick intervals of both mud and sand, in subequal amounts[84]. These cores have a higher sand content than those from the upper Congo Canyon[32], but are much muddier than cores from the axis of Monterey Canyon[85,87]. Sediment traps in the upper Gaoping Canyon recovered mainly muddy sediment (85% mud, 15% sand) [84]. The exact fraction of sand and mud in flows moving down Gaoping Canyon is thus uncertain, but it has a significantly higher mud content than Monterey Canyon, and a somewhat higher sand content than the Congo System.

Bute Inlet (~20% mud; ~80% sand). The submarine channel in this fjord is fed by the deltas of the Homothko and Southgate Rivers[58,63,64]. These braided rivers are dominated (~80%) by sandy bedload, with a subordinate amount (20%) of muddy suspended load that forms a surface plume[88]. The sediment input from both rivers combined is ~2-4 Mt/yr[63,64,80]. The axis of the submarine channel is typically entirely sand, as is the lobe at the end of the channel[63,80]. Cores from terraces have interbedded muds and sands, whilst deposits in the overbank areas and distal basin are almost exclusively mud[89]. Thus, turbidity currents originating from the river deltas are likely dominated by sand, albeit with more mud than occurs in Monterey Canyon. These flows in Bute Inlet probably also contain somewhat less mud than those within both Gaoping Canyon and Congo Canyon. However, it is again unclear how much sand and mud occur within the near-bed layers of these flows.

Comparison to other global sediment fluxes

Other annual global sediment mass fluxes are taken from the literature[6,68] (Table 1), but noting these mass fluxes most likely have even greater uncertainties than those for mass eroded in 2019-20 along the Congo Canyon-Channel. For example, many rivers are ungauged leading to significant uncertainties in suspended loads, and there is a near complete lack of measurements for riverine bedload, which is often assumed to be approximately 10% of the suspended load[6,68]. Measurements during extreme floods are especially rare.

For the Congo River, two estimates of annual suspended sediment flux are used. The first estimate is based on multi-decadal gauging station data in Kinshasa (29 Mt/yr)[44], although this flux may then vary before the river reaches its mouth. A single sampling campaign in November 1964 is the basis for a second estimate of 43 Mt/yr[55], which has been widely used in global reviews[6,68]. Field measurements in the braided region of the Congo River, close to its mouth, resulted in an estimate of ~130 Mt/yr for its bedload flux[54], which would greatly increase the total annual sediment load, and far exceed 10% of total sediment load that is often assumed for large rivers [6,68]. There are also large uncertainties for sediment fluxes in the Congo River.

SUPPLEMENTARY REFERENCES

71. Heezen, B.C., Menzies, R.J., Schneider, E.D., Ewing, M.W., & Granelip, C.L. Congo Submarine Canyon. *A.A.P.G. Bulletin*, 48, 1126-1149 (1964).

72. Bailey, L.P., Clare, M.A., Rosenberger, K., Cartigny, M.J.B., Talling, P.J., Paull, C.K., Gwiazda, R., Parsons, D.R., Simmons, S.M., Xu, J., Haigh, I.D., Maier, K.L., McGann, M., Lundsten, ER., & Monterey CCE Team. Preconditioning by sediment accumulation can produce powerful turbidity currents without major external triggers. *Earth and Planetary Sci. Letters* 562, art.116845 (2021).

73. Ralston, D. K., & Geyer, W. R. Sediment transport time scales and trapping efficiency in a tidal river. *J. Geophys. Res.* 122, 2042–2063 (2017).

74. Kineke, G.C., Sternberg, R.W., Trowbridge, J.H., & Geyer, W.R. Fluid-mud processes on the Amazon continental shelf. *Cont. Shelf Res.* 16, 667-696 (1996).

75. Parsons, J.D., Bush, J.W.M., & Syvitski, J.P.M. Hyperpycnal plume formation from riverine outflows with small sediment concentrations. *Sedimentology* 48, 465–478 (2001).

76. Hizzett, J. L., Hughes Clarke, J. E., Sumner, E. J., Cartigny, M. J. B, Talling, P. J., & Clare, M. A. Which triggers produce the most erosive, frequent and longest runout turbidity currents on deltas? *Geophys. Res. Letts,* 45, 855-863 (2017).

77. Mulder, T., Syvitski, J.P.M., Migeon, S., Faugeres, J.C., & Savoye, B. Marine hyperpycnal flows: Initiation, behavior and related deposits: A review: *Marine Petrol. Geol*. 20, 861-882 (2003).

78. Plink-Björklund, P., & Steel, R.J. Initiation of turbidity currents: outcrop evidence for Eocene hyperpycnal flow turbidites. *Sedim. Geol.* 165, 29–52 (2004).

79. Heerema, K., Cartigny, M.J.B., Silva Jacinto, R., Simmons, S.M., Apprioual, R. & Talling, P.J. 2021. How distinctive are flood-triggered turbidity currents? *J. Sedim. Res.* 92, 1–11 (2022).

80. Heijnen, M.S., Clare, M.A, Cartigny, M.J.B, Talling, P.J., Hage, S., Pope, E.L., Bailey, L., Sumner, E.J., Lintern, D.G., Stacey, C.D., Parsons, D.R., Simmons, S.M, Chen, Y., Hubbard, S.M., Eggenhuisen, J.T., Kane, I., & Hughes Clarke, J.E. Fill, flush or shuffle: How is sediment carried through submarine channels to build lobes? *Earth & Planet. Sci. Letters* 584, Art. 117481 (2022).

81. Hung, C., Lin, G-W., Kuo, H-L., Zhang, J-M., Chen, C-W., and Chen, H. Impact of an extreme typhoon event on subsequent sediment discharges and rainfall-driven landslides in affected mountainous regions of Taiwan. *Geofluids*, Article 8126518 (2018).

82. Chen, C-W., Oguchi, T., Hayakawa, Y.S., Saito, H., Chen, H., Lin, G-W., Wei, L-W., & Chao, Y-C. Sediment yield during typhoon events in relation to landslides, rainfall, and catchment areas in Taiwan. *Geomorphology* 303, 540-548 (2018).

83. Dadson, S.J., Chen, H., Dade, W.B., Hsieh, M.-L., Willett, S.D., Hu, J.-C., Horng, M.J., Chen, M.C., Stark, C.P., Lague, D., & Lin, J.-C. Links between erosion, runoff variability and seismicity in the Taiwan orogen. *Nature* 426, 648–651 (2003).

84. Liu, J.T., Hsu, R.T., Hung, J.J., Kao, S.J., Chang, Y.P., Huh, C.A.,Wang, Y.H., Lee, C.L., & Yang, R.J. From the highest to the deepest: the Gaoping River-submarine canyon dispersal system. *Earth Sci. Rev*. 153, 274-300 (2016).

85. Maier, K.L., Gales, J., Paull, C.K., Rosenberger, K., Talling, P.J., Simmons, S.M., Gwiazda, R.H., McGann, M., Cartigny, M.J., Lundsten, E., Anderson, K., Clare, M.A., Xu, J., Parsons, D., Barry, J.P., Wolfson-Schwher, M., Nieminski, N.M., & Sumner, E.J. Linking direct measurements of turbidity currents to submarine canyon-floor deposits. *Frontiers in Earth Science (Sed., Strat., Diag.)*. doi: 10.3389/feart.2019.00144 (2019).

86. Babonneau, N., Savoye, B., Cremer, M., & Bez, M. Sedimentary architecture in meanders of a submarine channel: detailed study of the present Congo turbidite channel (Zaiango Project). *J. Sedim. Res* 80, 852–866 (2010).

87. Symons, W.O., Sumner, E.J., Paull, C.K., Cartigny, M.J.B., Xu, J.P., Maier, K.L., Lorenson, T.D., & Talling, P.J. A new model for turbidity current behavior based on integration of flow monitoring and precision coring in a submarine canyon. *Geology* 45, 367-370 (2017).

88. Syvitski, J. P. M., & Farrow, G. E. Structures and processes in Bayhead Deltas: Knight and Bute Inlet, British Columbia. *Sedim. Geol. 36*, 244 (1983).

89. Heerema, C. J. Evolution of turbidity currents: new insights from direct field measurements, Durham theses, Durham University. *Available at Durham E-Theses Online:* http://etheses.dur.ac.uk/13963/ (2021).

90. Chiang, L-C., Wang, Y-C., & Liao, C-J. Spatiotemporal variations of sediment export from multiple Taiwan watersheds. *Int. J. Environ. Res. Public Health* 2019, 16, 1610 (2019).

91. Gwiazda, R., Paull, C.K., Kieft, B., Klimov, D., Herlien, R., Lundsten, E., McCann, M., Cartigny, M.J.B., Hamilton, A., Xu, J., Maier, K.L., Parsons, D.R., & Talling P. J. Near-bed structure of sediment gravity flows measured by motion-sensing 'boulder-like' Benthic Event Detectors (BEDs) in Monterey Canyon. *J. Geophys. Research*. 127(2) doi:10.1029/2021JF006437 (2022).

SUPPLEMENTARY TABLE 1: Time of faults (2002-2022) on seabed cables due to turbidity currents along Congo Canyon.

Cable	Cable Fault - Date and Time	Latitude	Longitude	Water Depth (m)	Distance from shore (km)
SAT-3 cable operational from 2001, WACS cable from 2012, and ACE cable from 2021					
SAT-3	01.23 UTC on 9th Oct. 2002	5.946S	9.416 E	3573	552
January 14-16th 2020 flow					
WACS (shallow)	No fault occurred	5.644 S	10.659 E	2584	262
SAT-3	23:05 UTC on 15th Jan. 2020	5.946S	9.416 E	3573	552
WACS (deeper)	07:54 UTC on 16th Jan. 2020	5.722 S	8.303 E	4098	773
March 9th 2020 flow					
WACS (shallow)	No fault occurred	5.644 S	10.659 E	2584	262
SAT-3	9th March (no exact time)	5.946S	9.416 E	3573	552

	Broke same place as Jan 15th.				
WACS (deeper)	No fault occurred	5.722 S	8.303 E	4098	773
April 28-29th 2021 flow					
WACS (shallow)	No fault occurred	5.644 S	10.659 E	2584	262
SAT-3	15.06 UTC on 28th April 2021	5.946S	9.416 E	3573	552
WACS (deeper)	No fault occurred	5.722 S	8.303 E	4098	773
ACE	15.02 UTC on 29th April 2021	6.09 S	7.57 E	4368	897
January 28th 2022 flow					
WACS (shallow)	No fault occurred	5.644 S	10.659 E	2584	262
SAT-3	16.36 UTC on the 28th January	5.946S	9.416 E	3573	552
WACS	No fault occurred	5.722 S	8.303 E	4098	773
ACE	January 28th (time uncertain)	6.09 S	7.57 E	4368	897

SUPPLEMENTARY TABLE 2.

Arrival times and distances between moorings and cables used to calculate flow transit speeds.

Mooring Name	Position - Latitude (DDM)	Position - Longitude (DDM)	date mooring starts recording (day after 11 sept)	date mooring stops recording and surfaced	Water depth (m)	Distance from shore (m)
M3	5 57.21 S	11 33.19 E	20 Sept (Day 9)	10-Oct-19	1565	101,999
M4	5 55.45 S	11 28.41 E	20 Sept (Day 9)	15-Jan-20	1629	113,361
M1	5 54.01 S	11 19.91 E	19 Sept (Day 8)	10-Oct-19	1875	136,180
Aniitra2	5 54.01 S	11 19.68 E	19 Sept (Day 8)	29-Dec-19	1875	136,417
Aniitra3	5 52.73 S	11 10.5 E	21 Sept (Day 10)	10-Oct-19	2017	163,495
M2	5 50.23 S	11 2.33 E	25 Sept (Day 14)	15-Jan-20	2172	190,597
M9	5 41.18 S	10 42.58 E	21 Sept (Day 10)	15-Jan-20	2172	190,918
SAT3 submarine cable					3573	552,415
WACS submarine cable					~3800	772,685
M5	5 43.87 S	8 7.32 E	28 Sept (Day 17)	15-Jan-20	4036	797,870
M8	5 45.41 S	7 40.49 E	2 Oct (Day 21)	15-Jan-20	4299	877,199
M6	5 52.14 S	6 55.52 E	29 Sept (Day 18)	15-Jan-20	4495	992,891
M7	6 27.86 S	6 2.83 E	11 Sept (Day 0)	15-Jan-20	4736	1,128,339

Mooring Name	Oct 10 Event	Oct 10 Event - speed	27-28 Oct Event	27-28 Oct Event speed	30 Oct Event	30 Oct Event - speed
M3	'10-Oct-2019 02:42:36'		no data		no data	
		M3>M1: 34181m in 8892s = 3.84 m/s (+/-0.08 m/s)				
M4	no data		no data		no data	
M1	'10-Oct-2019 05:10:48'		no data		no data	
Aniitra2	'10-Oct-2019 05:09:45'		'27-Oct-2019 14:45:45'		30-Oct-2019 02:36:45'	
		A2>M2: 54180m in 29484s = 1.84 m/s (+/- 0.04 m/s)				
Aniitra3	no data		no data		no data	A2>M2: 54180m in 47998s = 1.13 m/s (+/- 0.02)
				A2>M2: 54180m in 75327s = 0.72 m/s (+/- 0.01)		
M2	'10-Oct-2019 13:21:09'		'28-Oct-2019 11:41:42'		'30-Oct-2019 15:56:33'	
M9	'10-Oct-2019 13:25:54'	M2>M9:321m in 285s = 1.13 m/s (+/- 0.10 m/s)	28-Oct-2019 11:50:58'	M2>M9: 321m in 556s = 0.58 m/s (+/- 0.03)	no data	
SAT3 submarine cable	no break	no break	no break	no break	no break	no break
WACS submarine cable	no break	no break	no break	no break	no break	no break
M5	no flow recorded	no flow recorded	no flow recorded	no flow recorded	no flow recorded	no flow recorded
M8	no flow recorded	no flow recorded	no flow recorded	no flow recorded	no flow recorded	no flow recorded
M6	no flow recorded	no flow recorded	no flow recorded	no flow recorded	no flow recorded	no flow recorded
M7	no flow recorded	no flow recorded	no flow recorded	no flow recorded	no flow recorded	no flow recorded

Mooring Name	24-25 Nov Event	24-25 Nov Event speed	28-29 Nov Event	28-29 Nov Event - speed	15-16 Dec Event 10 -	15-16 Dec Event - speed
M3	no data		no data		no data	
M4	no data		no data		no data	
M1	no data		no data		no data	
Aniitra2	'24-Nov-2019 18:10:30'		'28-Nov-2019 10:17:14'		'15-Dec-2019 01:17:15'	
Aniitra3	no data	A2>M2: 54180m in 22554 s = 2.40 m/s (+/-0.05 m/s)	no data	A2>M9: 54501m in 209168s = 0.26 m/s (+/-0.01 m/s)	no data	A2>M9: 54501m in 94759s = 0.58 m/s (+/- 0.01 m/s)
M2	'25-Nov-2019 00:26:24'		no data		no data	
M9	'25-Nov-2019 00:28:55'	M2>M9: 321m in 151s = 2.13 m/s (2.49-1.84)	'29-Nov-2019 15:23:22'		'16-Dec-2019 03:36:34'	
SAT3 submarine cable	no break	no break	no break	no break	no break	no break
WACS submarine cable	no break	no break	no break	no break	no break	no break
M5	no flow recorded	no flow recorded	no flow recorded	no flow recorded	no flow recorded	no flow recorded
M8	no flow recorded	no flow recorded	no flow recorded	no flow recorded	no flow recorded	no flow recorded
M6	no flow recorded	no flow recorded	no flow recorded	no flow recorded	no flow recorded	no flow recorded
M7	no flow recorded	no flow recorded	no flow recorded	no flow recorded	no flow recorded	no flow recorded

Mooring Name	27 Dec Event	27 Dec Event - speed	8-15 Jan Event	8-15 Jan Event speed	Jan 14-16 Event (Big flow, Broke cables)	Jan 14-16 Event - speeds
M3	no data		no data		no data	
M4	no data		no data		Jan 14th 2020 22:30:51	
						M4>M2: 77,236m in 14,657 = 5.27 m/s (+/- 0.10 m/s)
M1	no data		no data			
Aniitra2	'27-Dec-2019 18:20:15'		no data		no data	
Aniitra3	no data	A2>M9: 54180m in 16749s = 3.23 m/s (+/- 0.06)	no data		no data	
M2	no data		no data		'15-Jan-2020 02:56:42'	
M9	'27-Dec-2019 23:00:11'		'08-Jan-2020 12:14:01'		'15-Jan-2020 02:56:43'	
						M9>SAT-3: 361,497m in 72,497s = 4.99 m/s (+/- 0.1 m/s)
SAT3 submarine cable	no break	no break	no break		cable break @ 23:05 on 15th Jan	
				M9>M5: 606952m in 137843s = 4.40 m/s (+/- 0.09)		SAT-3>WACS: 220,270m in 31,740 = 6.94 m/s (+/- 0.14 m/s)
WACS submarine cable	no break	no break	no break		cable break @ 07:54 on 16th Jan	
						WACS>M5: 25185m in 3564s = 7.07m/s (+/-0.14 m/s)

M5	no flow recorded	no flow recorded	'10-Jan-2020 02:31:24'		'16-Jan-2020 08:53:24'	
				M5>M8: 79329m in 176128s = 0.45 m/s (+/- 0.01)		M5>M8R: 79329m in 9,796s = 8.10 m/s (+/- 0.16 m/s)
M8	no flow recorded	no flow recorded	'12-Jan-2020 03:26:52'		'16-Jan-2020 11:36:40'	
				M8>M6: 115692m in 314768s = 0.37 m/s (+/-0.01)		M8R>M6: 115692m in 17480s = 6.62 m/s (+/- 0.13 m/s)
M6	no flow recorded	no flow recorded	'15-Jan-2020 18:53:00'		'16-Jan-2020 16:28:00'	
						M6>M7: 135448m in 16407s = 8.26 m/s (+/-0.17 m/s)
M7	no flow recorded	no flow recorded	no flow recorded	flow never reached last mooring	'16-Jan-2020 21:01:27'	

SUPPLEMENTARY TABLE 3. Summary of key parameters for 4 locations where detailed measurements are available for changes in front speed with distance for turbidity currents confined in canyon-channels (Fig. 7).

Key Parameter for flows and systems	Congo Canyon, offshore W. Africa	Monterey Canyon, off California, USA	Gaoping Canyon, Offshore Taiwan	Bute Inlet, British Columbia, Canada
Data type for front speed changes	ADCP-moorings and cable break timing[9]	ADCP Moorings only[34]	Cable breaks only[24,25]	ADCP Moorings only[59]
Threshold front speed for ignition or autosuspension	4-5 m/s (Fig. 6a)	4-5m/s[35] (fig. 6b)	> 5 m/s (fig. 6c) Possibly lower	4-5 m/s (fig. 6d)
Flow runout distance	> 1,100 km (Fig. 5a)	< 50-70 km (Fig. 5b) [34,35]	> 350-400 km (Fig. 5d) [24,25]	< 50 km (fig. 5d) [59]
Depth of erosion and deposition	Often 20-30 m erosion, but little deposition (Fig. 7) over 12 months, by two main flows	Sub-equal areas of erosion/ deposition, depths <2-3 m; linked to migrating bedforms[34,91]	Unknown - no repeat surveys	Alternating areas of erosion/ deposition, depths +/- 25m, due to knickpoints that migrate ~400m/yr[63]
Volume of erosion and deposition	2.68 km^3 [>1.00 km^3] (~1,300-2,600 Mt) (via two main flows)	Not available	Unknown	~3.7 Mt/yr erosion; and 1.7 Mt/yr deposition[80]
Sand v. mud ratio (Supplement. Disc.)	> 60% mud <40% sand	<10% mud >90% sand	> 50% mud (?) < 50% sand (?)	~20% mud ~80% sand
Seabed gradient range	Declining from 0.6° to 0.3° (Fig. 5c)	Near uniform at 1.4° (Fig. 6b) [35]	Declining from 0.4° - 0.3° (Fig. 6c) [25]	Initially 4°, but mainly ~0.4°; higher at knickpoints [58,63,64]
Canyon-channel width range	1 to 1.5 km (first terrace or levee)	150-200 m, but widening to 700 m[35]	Poorly known	150-200 m
Input sediment flux	29-43 Mt/yr average suspended load [6,44] (and another 130 Mt/yr in bedload? [54])	1.2 Mt/yr (mainly as bedload) [72]	40 Mt/yr average of suspended load[84]; But 280 - 570 Mt in Typhoon Morakot [81,82,90]	~4 Mt/yr (including suspended and bedload[63,80,88]
Sediment source	Congo River (homopycnal) [52]	Longshore drift[72]	Gaoping River[84] (sometimes hyperpycnal)	Homothko and Southgate Rivers (homopycnal) [63,80,88]
Flow triggers	River floods and spring tides	Large storm waves; but not always[72]	Major earthquakes and major typhoons	River floods and tidal cycles

SUPPLEMENTARY TABLE 4. Volumes of seabed erosion and deposition calculated using different methods and limits of detection (see Supplementary Discussion, Supplementary Figs. 7-10, and Schimel et al. [69]).

Method used	Seabed change volume: Upper Canyon survey (Angolan waters)	Seabed change volume: Lower Channel survey (international waters)	Seabed change volume: Total (both surveys - 40% of total system length)
No limit of detection (data from all grid cells are used)	-0.299 km^3 (-0.319 km^3 erosion; +0.020 km^3deposition)	-0.768 km^3 (-1.081 km^3 erosion; +0.313 km^3 deposition)	-1.066 km^3 (-1.390 km^3 erosion; +0.333 km^3 deposition)
Spatially variable CUBE limit of detection (k = 1)	-0.274 km^3 (-0.280 km^3 erosion; +0.006 km^3 deposition)	-0.445 km^3 (-0.507 km^3 erosion; +0.062 km^3 deposition)	-0.718 km^3 (-0.786 km^3 erosion; +0.068 km^3 deposition)
Spatially variable CUBE limit of detection (k = 1.96)	-0.203 km^3 (-0.201 km^3 erosion; +0.002 km^3 deposition)	-0.192 km^3 (-0.212 km^3 erosion; +0.020 km^3 deposition)	-0.395 km^3 (-0.417 km^3 erosion; +0.022 km^3 deposition)
Spatially fixed limit of detection that is 5 m (in upper canyon) or 15 m (in lower channel survey)	-0.282 km^3 (-0.289 km^3 erosion; +0.007 km^3 deposition)	-0.347 km^3 (-0.391 km^3 erosion; +0.044 km^3 deposition)	-0.628 km^3 (-0.679 km^3 erosion; +0.051 km^3 deposition)

SUPPLEMENTARY FIGURES

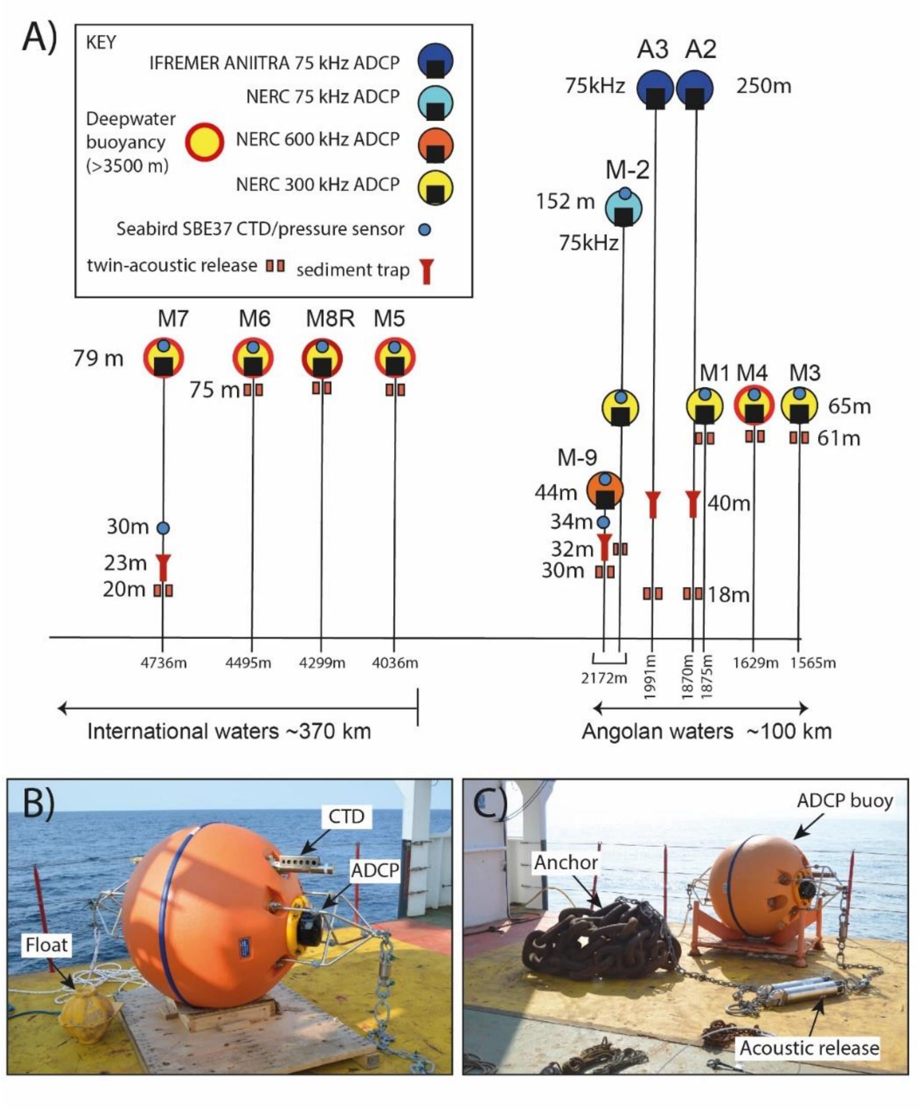

Supplementary Figure 1. (A) Summary diagram of the array of 11 moorings deployed along the Congo Canyon and Channel in 2019-2020 (locations in Fig. 1)[9]. Seven moorings were deployed in shallow, Angolan waters in the upper canyon (right side), and four moorings were deployed in deeper, international waters (left side). The diagram shows the type of ADCP and other sensors included in each mooring, and their heights above the seabed. Water depths of mooring anchors are shown, together with positions of sediment traps, and acoustic releases for recovery. However, all moorings surfaced after their wire was broken by turbidity currents, on the various dates shown in Figure 2. **(B and C)** Photographs of moorings on deck before deployments in 2019, showing the anchor, buoyant float that houses the ADCP and CTD sensors, and the two acoustic releases, which are all linked via chain or wire.

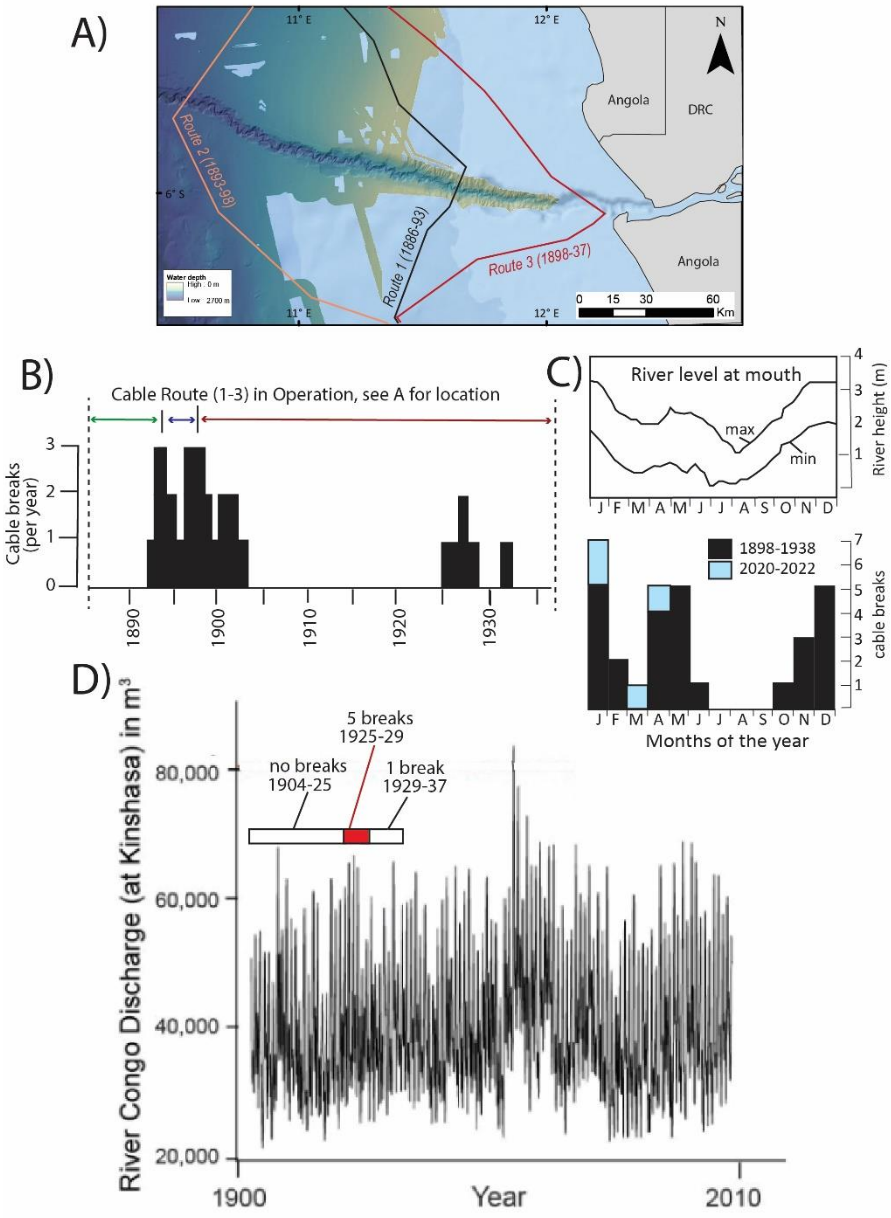

Supplementary Figure 2. Insights into timing, frequency and triggers of turbidity currents in upper Congo Canyon from older (1886 to 1937) seabed cable breaks[71]. These cable routes were located in the upper canyon, closer to shore than the currently active telecommunication cables (Fig. 1). **(A)** Map of upper canyon and river mouth showing three cable routes used from 1886-1893, 1893-1898, and 1898-1937[71]. **(B)** Number of cable breaks each year between 1886 to 1937 (black) and 2020-22 (blue). **(C)** Monthly changes in Congo River level at a gauging station near its mouth, and total number of cable breaks in each month, from 1989 to 1938. **(D)** Changes in annual maximum discharge of the Congo River at Kinshasa from 1900-2008, compared to periods in which there were five cable breaks red box (1900-1904), no cable breaks (1904-1923), 5 cable breaks (1925-1929), and one cable break (1928-1937). Parts A-C after[71].

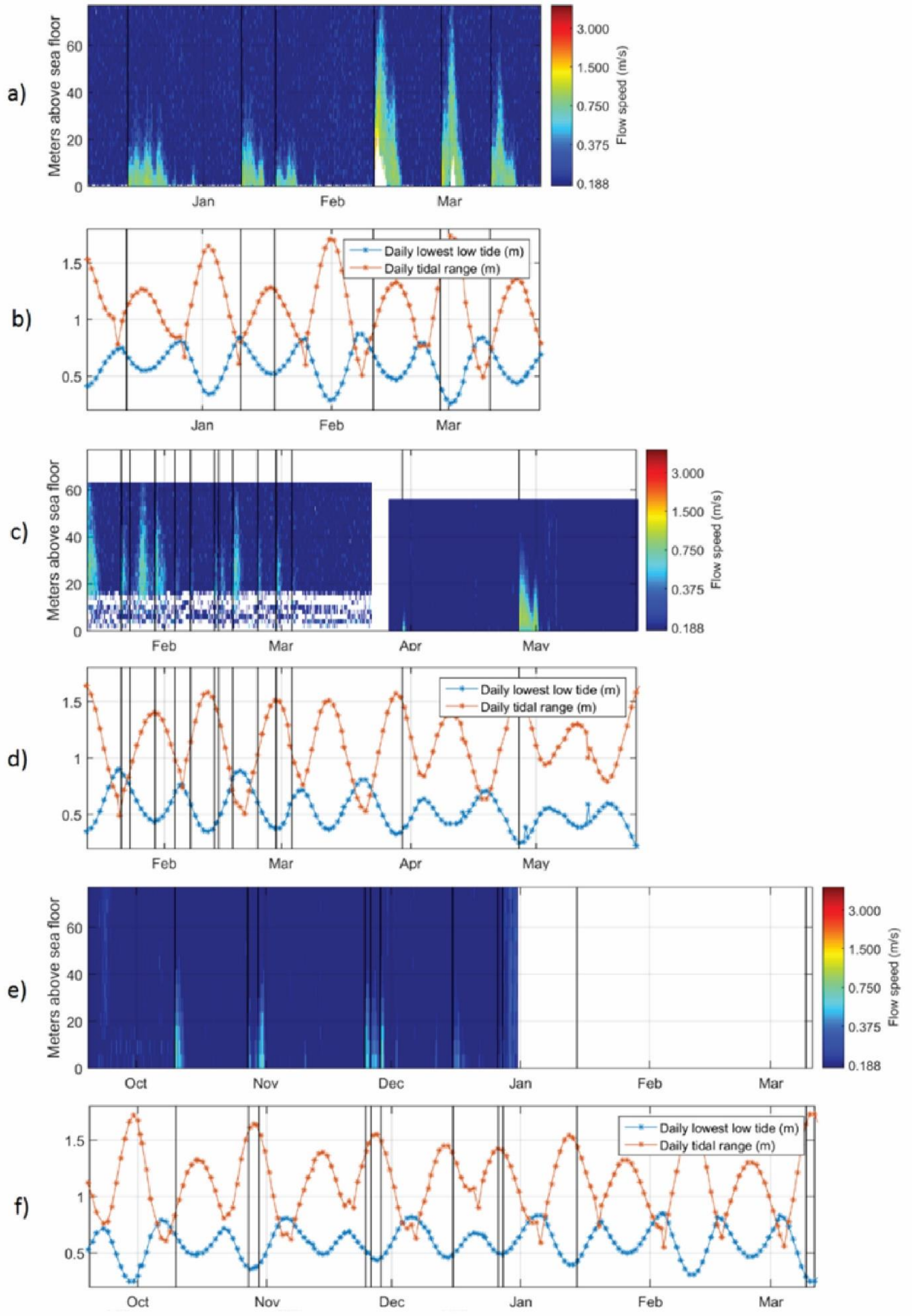
a)
Meters above sea floor
Jan
Feb
Mar
Flow speed (m/s)
3.000
1.500
0.750
0.375
0.188
b)
Daily lowest low tide (m)
Daily tidal range (m)
c)
Apr
May
d)
e)
Oct
Nov
Dec
f)

Supplementary Figure 3. Relationship between the timing of smaller flows recorded by ADCPs in the upper canyon and tidal cycles in 2009-10, 2013 and 2019-20. For each period, velocity profile data from a moored ADCP in the upper canyon are compared to tidal data from Soyo in the Congo River mouth, with vertical bars indicating flow arrival times in tidal time series. **(a and b)** Timing of turbidity currents and tidal cycles in 2009-10. **(c and d)** Timing of turbidity currents and tidal cycles in 2013, with ADCP data shown from two different mooring deployments. **(e and f)** Timing of turbidity currents and tidal cycles in 2019-20. ADCP-moorings were damaged and returning no data after a large flow on January 14th. Also shown is timing of a turbidity current on March 9th 2020 based on cable breaks. There is a delay between flow initiation at river mouth, and arrival at these offshore mooring that are ~100 km from the river mouth. This time delay is strongly dependent on flow front speed. For example, the delay is ~0.35 days for front speeds of 5 m/s, 0.86 days at 2 m/s, 1.73 days at 1 m/s, 3.46 days at 0.5 m/s, and 8.65 days at 0.2 m/s. Thus, time delays start to the approach the duration of spring-neap tidal cycles for slower flows, and this makes it difficult to compare the time at which slower flows started with spring-neap tidal cycles. Thus, only the timing of faster (>2-4 m/s) turbidity currents can be compared reliably to tidal cycles.

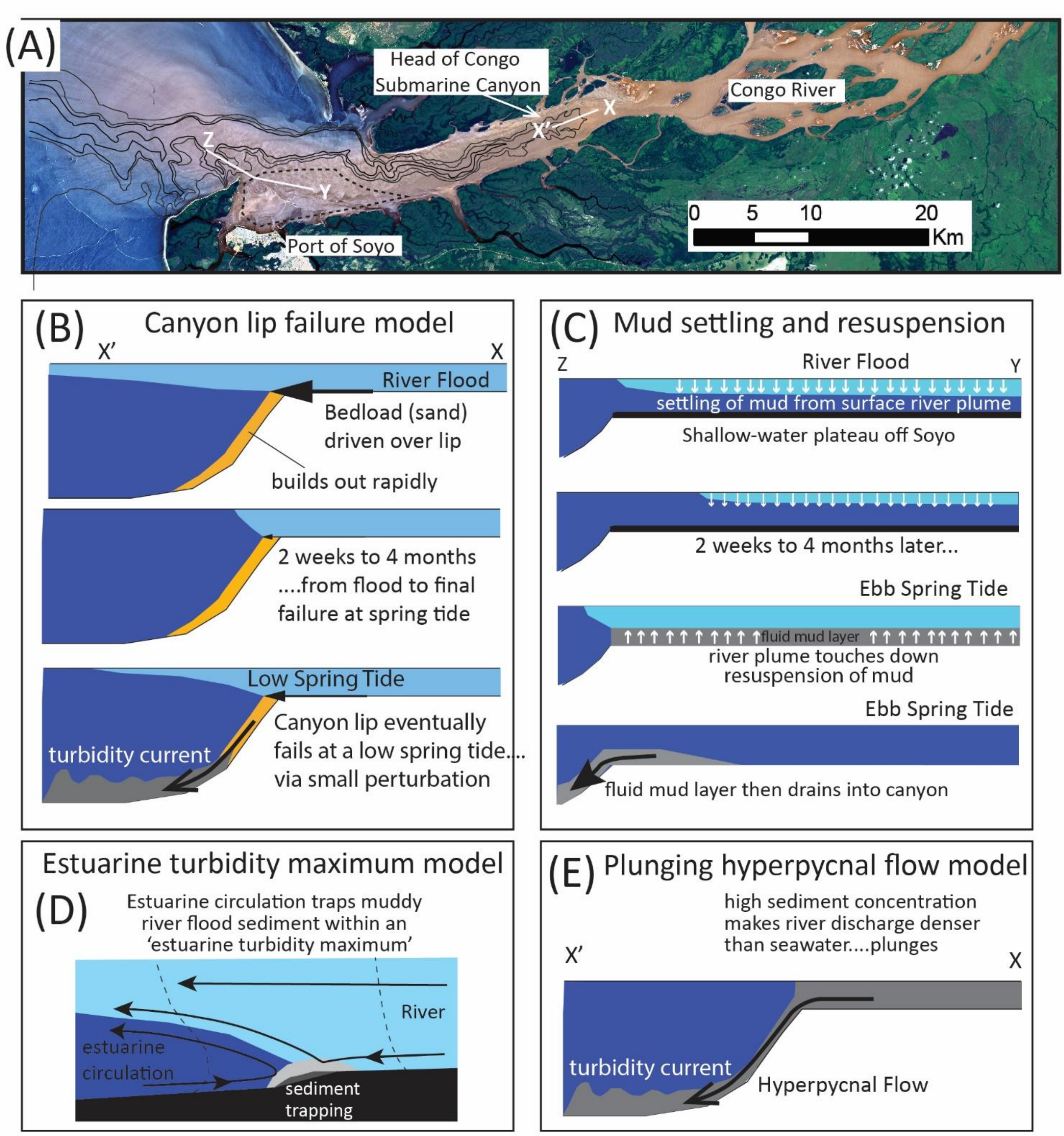

Supplementary Figure 4. Different models for initiation turbidity currents due to an initial river flood, but finally triggered at a spring tide. **(a)** Satellite image of the Congo River and the estuary at its mouth, with bathymetric contours (Fig. 1d). Image also shows main head of the submarine canyon (X-X), and a shallow water plateau offshore Soya (Y) that lies next to tributary canyon heads (Z). **(b)** Model 1: flood drives sediment over the lip of the submarine canyon, which fails weeks to months later at a low spring tide, to generate a major turbidity current. **(c)** Model 2: mud settles from the surface plume (part a) onto the seabed, and is then resuspended at a spring tide to form a highly mobile fluid mud layer, which then drains

into the tributary canyon heads to generate a turbidity current. **(d)** Model 3: muddy sediment is trapped within the estuary due to near-bed estuarine circulation - in an estuarine turbidity maximum. **(e)** Model 4: hyperpycnal flow model in which the river discharge has sufficient suspended sediment to be denser that seawater, such that the river discharge plunges to flow directly along the seabed.

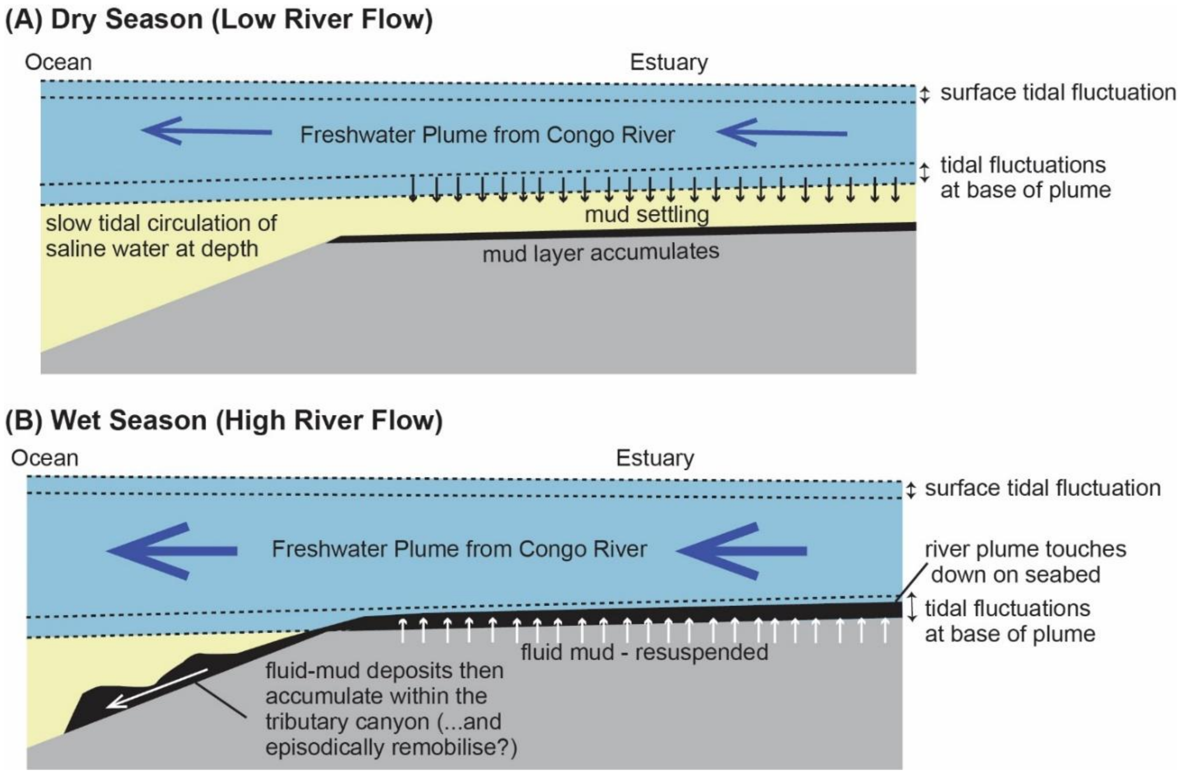

Supplementary Figure 5. Schematic summary of field observations for a transect across the extensive shallow-water plateau upstream from Soyo to the tributary canyon heads (Y-Z in Supplementary Fig. 4a), in a year without an exceptional river flood (R. Nunny, *pers. comm.,* 2021). **(a)** During months of low river flow in a normal (non-flood) year, mud settles from the freshwater surface river plume, forming a mud layer across the plateau. **(b)** During months with high river flow in a normal year, the surface river plume touches down on the seabed across the plateau, especially during spring tides. This acts to remobilise the seabed mud, thereby forming highly-mobile fluid-mud layers that can be several meters thick. These fluid mud layers then move into the tributary canyon head, where deposit mud within the tributary canyon. However, the mud deposit in the tributary canyon may then episodically remobilise to form unusually large turbidity currents that runout for even longer distances.

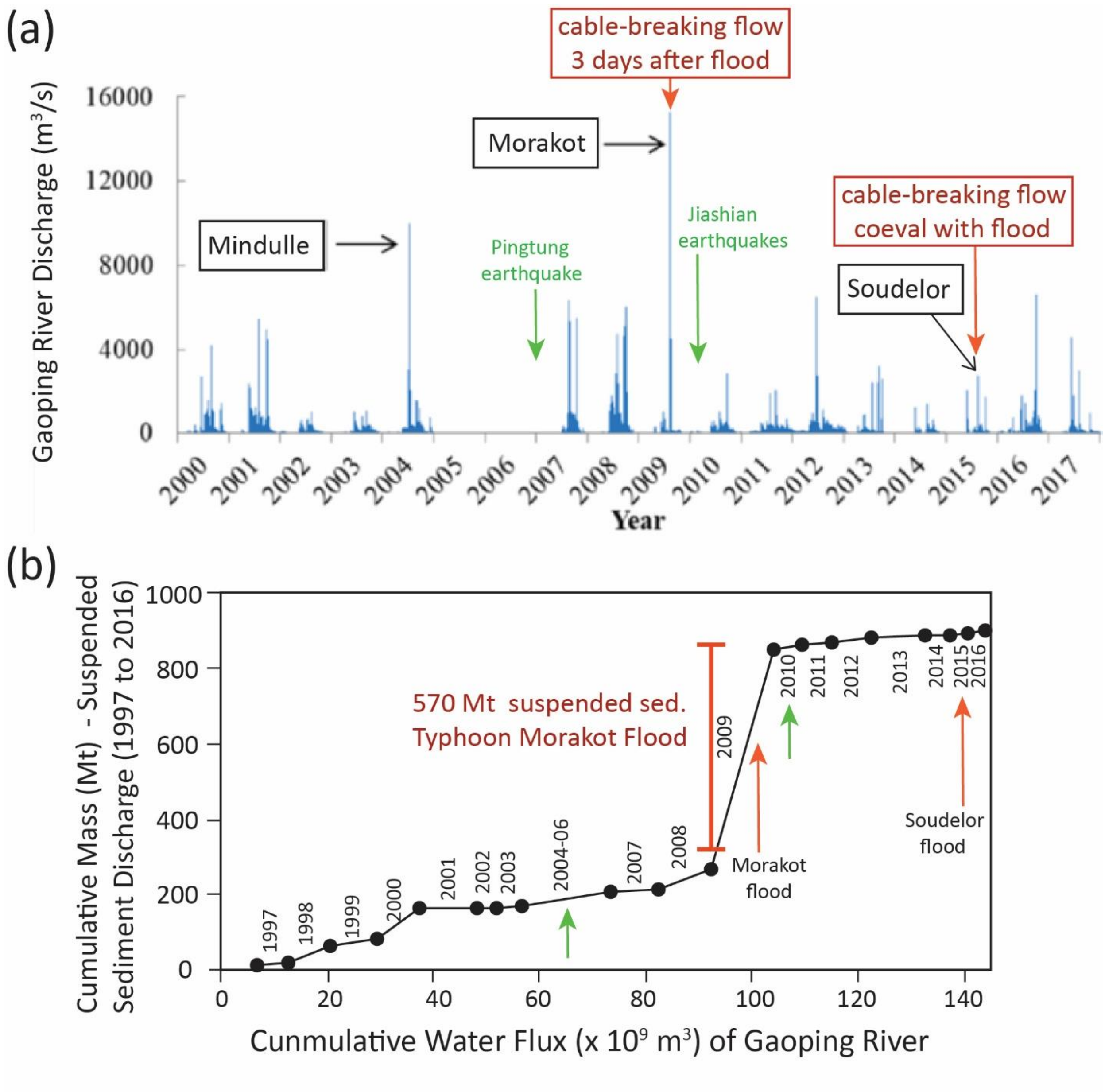

Supplementary Figure 6. Relationships between Gaoping River water and suspended sediment discharge, and timing of powerful and long runout (cable-breaking) turbidity currents in Gaoping Canyon. **(a)** Gaoping River water discharge from 2000-2016 showing timing of powerful offshore turbidity currents linked to river floods caused by Typhoons Morakot in 2009 and Typhoon Soudelor in 2015 (red arrows), or the Pingtung (2006) and Jiashian (2010) earthquakes (green arrows). Modified from Chiang et al. (2019)[90]. (b) Changes in cumulative water and suspended sediment flux in the Gaoping River through time. The powerful turbidity current in 2009 was linked to an exceptional flood that supplied ~570 Mt of suspended sediment (~15 years of the average supply) to the lower part of the Gaoping River. In contrast, the powerful offshore flow in 2015 coincided with a far smaller flood along the Gaoping River. Modified from Hung et al. (2018) [81].

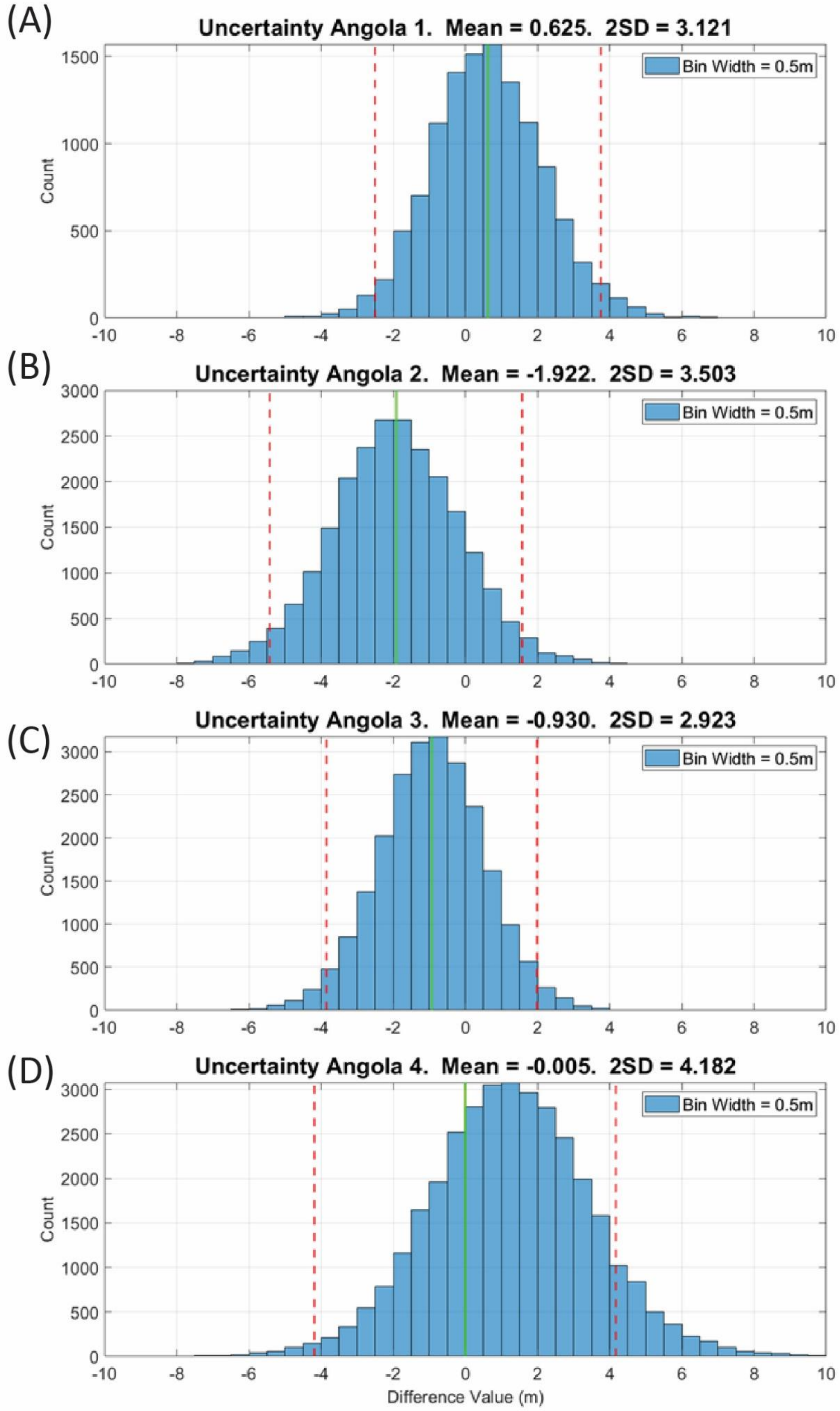
(A)
Uncertainty Angola 1. Mean = 0.625. 2SD = 3.121
Bin Width = 0.5m
Count
(B)
Uncertainty Angola 2. Mean = -1.922. 2SD = 3.503
Bin Width = 0.5m
Count
(C)
Uncertainty Angola 3. Mean = -0.930. 2SD = 2.923
Bin Width = 0.5m
Count
(D)
Uncertainty Angola 4. Mean = -0.005. 2SD = 4.182
Bin Width = 0.5m
Count
Difference Value (m)

(E)

(F)

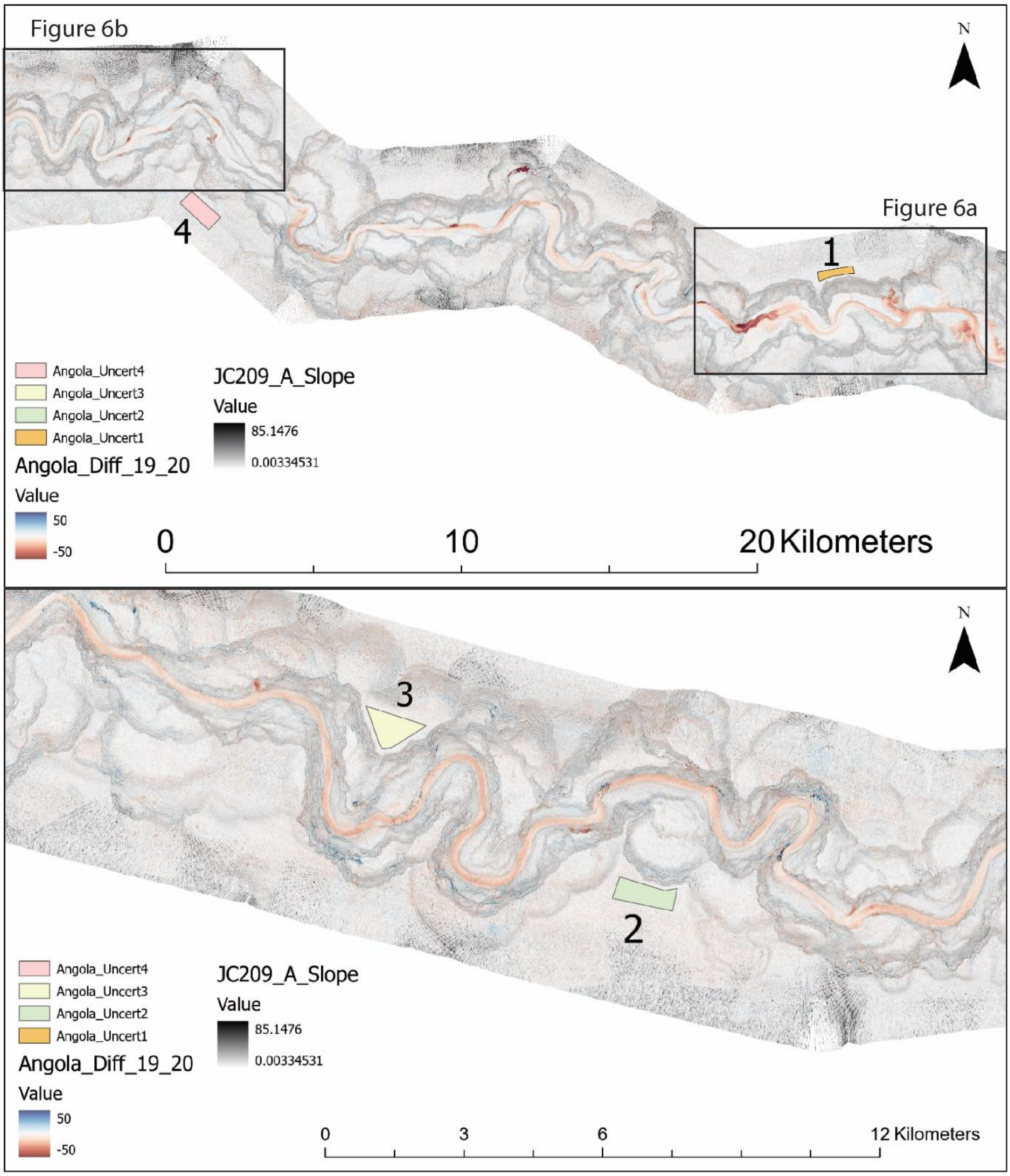

Supplementary Figure 7. Measured differences in seabed elevation for areas outside the main axis of the upper canyon from 2019-20, where it is assumed that no significant (< 0.5 m) changes actually occurred. Plots provide a method for assessing uncertainties and limits for detection for seabed change in the upper canyon (see Methods section in main text). **(a-d)** Histograms of changes in seabed elevation (m) for four areas located outside the axis of the upper canyon in Angolan Waters, at sites shown by numbered rectangles shown in part. The differences in seabed elevation from 2019-20 are typically less than 5 meters (Supplementary Table 4). **(e-f)** Maps showing location of the four areas used to generate data in parts A-D.

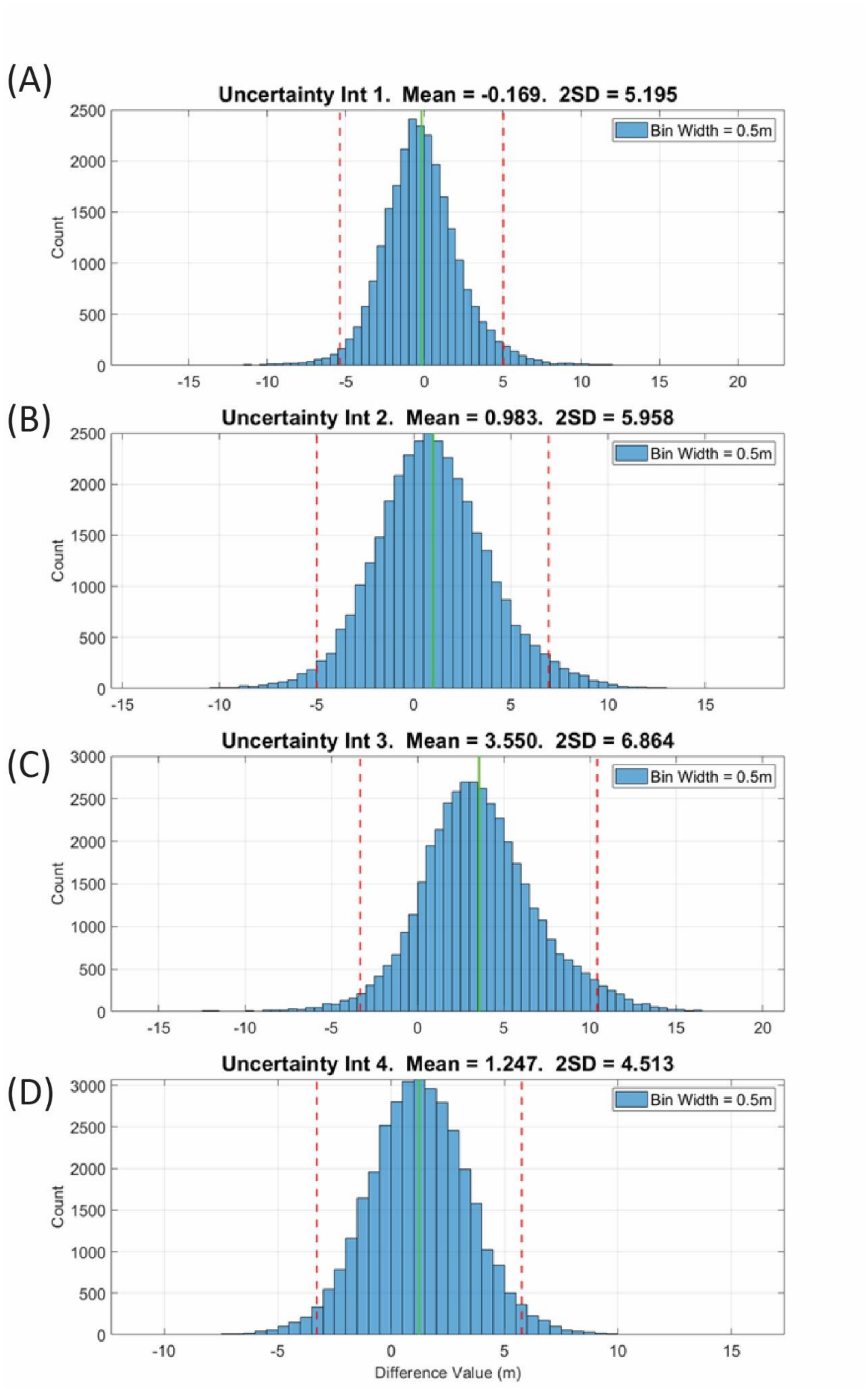
(A)
Uncertainty Int 1. Mean = -0.169. 2SD = 5.195
Bin Width = 0.5m
Count
(B)
Uncertainty Int 2. Mean = 0.983. 2SD = 5.958
Bin Width = 0.5m
Count
(C)
Uncertainty Int 3. Mean = 3.550. 2SD = 6.864
Bin Width = 0.5m
Count
(D)
Uncertainty Int 4. Mean = 1.247. 2SD = 4.513
Bin Width = 0.5m
Count
Difference Value (m)

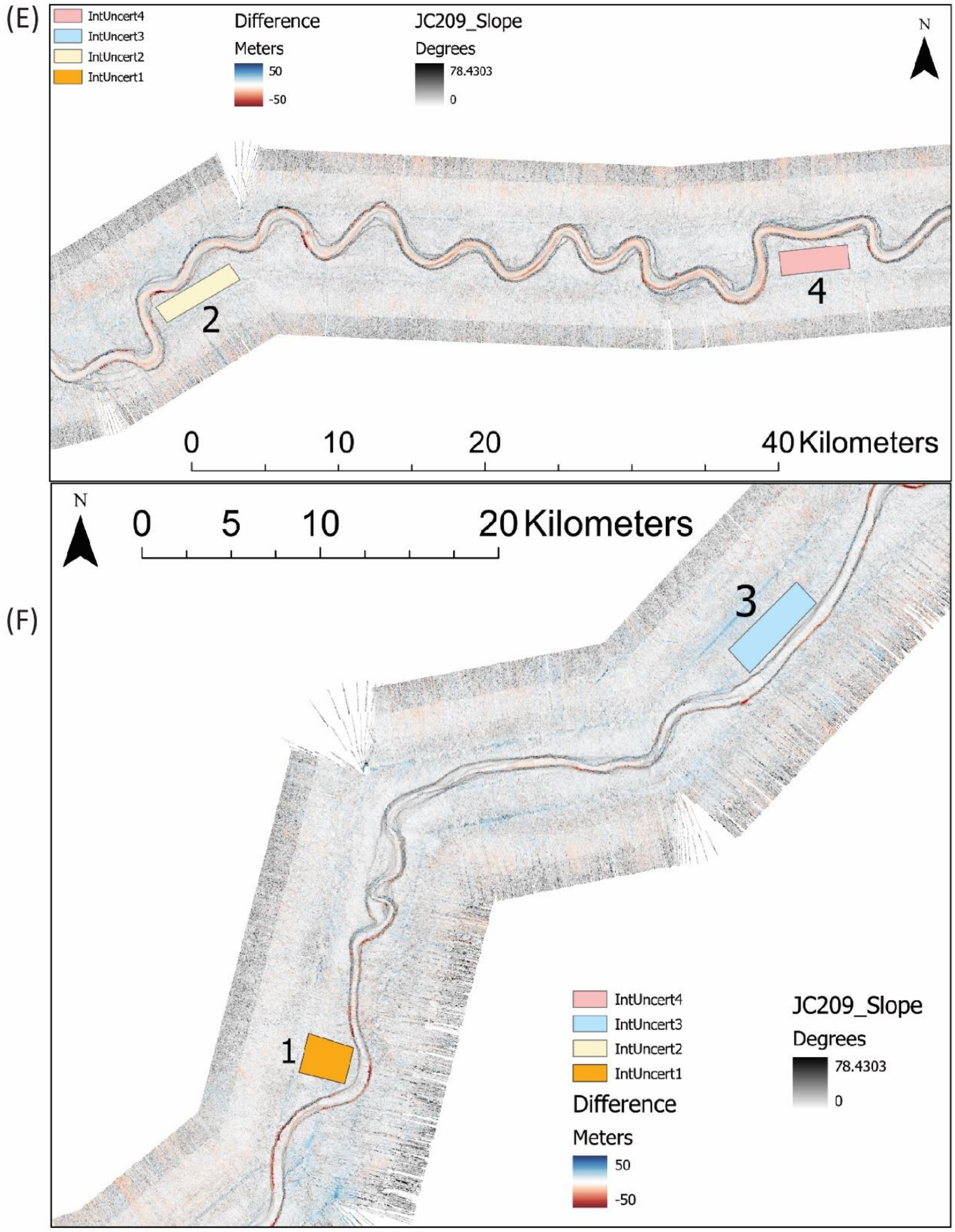

Supplementary Figure 8. Measured differences in seabed elevation for areas outside the main axis of the lower channel from 2019-20, where it is assumed that no significant (< 0.5 m) changes actually occurred. Plots provide a method for assessing uncertainties and limits for detection for seabed change in the lower channel (see Methods section). **(a-d)** Histograms of changes in seabed elevation (m) for four areas located outside the axis of lower channel in International Waters, at sites shown by numbered rectangles shown in part. The differences in seabed elevation from 2019-20 are typically less than 10 meters (Supplementary Table 4). **(e-f)** Maps showing location of the four areas used in parts A-D.

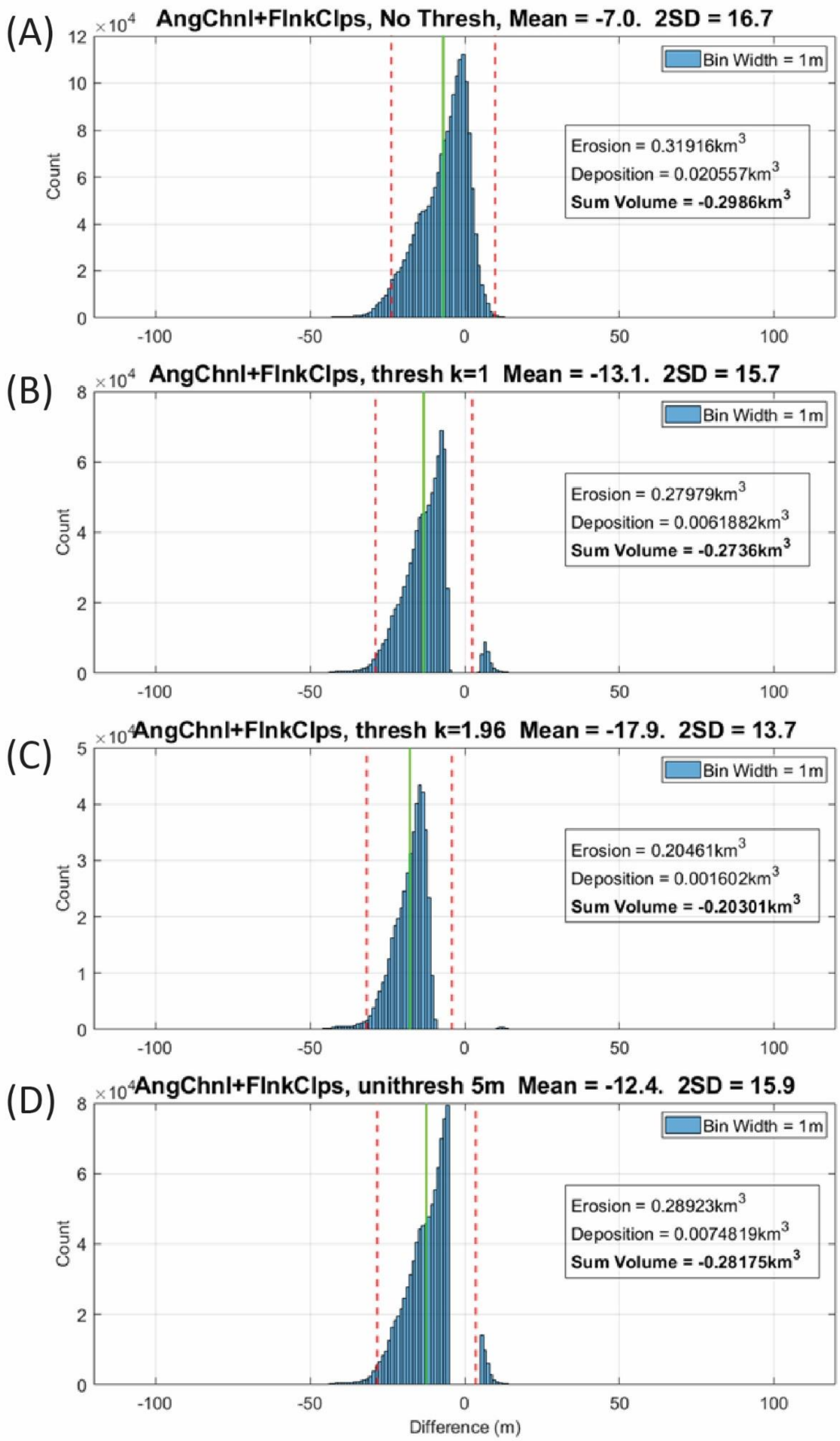

(A)
AngChnl+FlnkClps, No Thresh, Mean = -7.0. 2SD = 16.7
Bin Width = 1m
Erosion = 0.31916km^3
Deposition = 0.020557km^3
Sum Volume = -0.2986km^3
(B)
AngChnl+FlnkClps, thresh k=1 Mean = -13.1. 2SD = 15.7
Bin Width = 1m
Erosion = 0.27979km^3
Deposition = 0.0061882km^3
Sum Volume = -0.2736km^3
(C)
AngChnl+FlnkClps, thresh k=1.96 Mean = -17.9. 2SD = 13.7
Bin Width = 1m
Erosion = 0.20461km^3
Deposition = 0.001602km^3
Sum Volume = -0.20301km^3
(D)
AngChnl+FlnkClps, unithresh 5m Mean = -12.4. 2SD = 15.9
Bin Width = 1m
Erosion = 0.28923km^3
Deposition = 0.0074819km^3
Sum Volume = -0.28175km^3
Count
Difference (m)

Supplementary Figure 9. Different methods for estimating uncertainties and limits of detection in seabed change along the upper canyon (Angolan territorial waters) (for location see Fig. 1). Plots show histograms of difference in seabed elevation change at individual grid cells along the axis of the Congo Canyon-channel system, between multibeam sonar surveys in September-October 2019 and October 2020. The modal elevation change (green line) and two standard deviations (red lines) are noted, along with the total volume of erosion in survey area (Supplementary Table 4). **(a)** All values of seabed change from every grid cell, without any limit of detection. **(b)** Grid cells removed if below a spatially varying limit of detection based on CUBE algorithm and k = 1 (raw CUBE-derived uncertainties). **(c)** Grid cells removed if below a spatially varying limit of detection based on the CUBE algorithm and k = 1.96 (i.e. 1.96 x raw CUBE-derived uncertainties) as in Mountjoy et al.[20]. **(d)** Grid cells removed if below a spatially fixed limit of detection set at 5 m. See Schimel et al.[69] for a detailed discussion of these different methods.

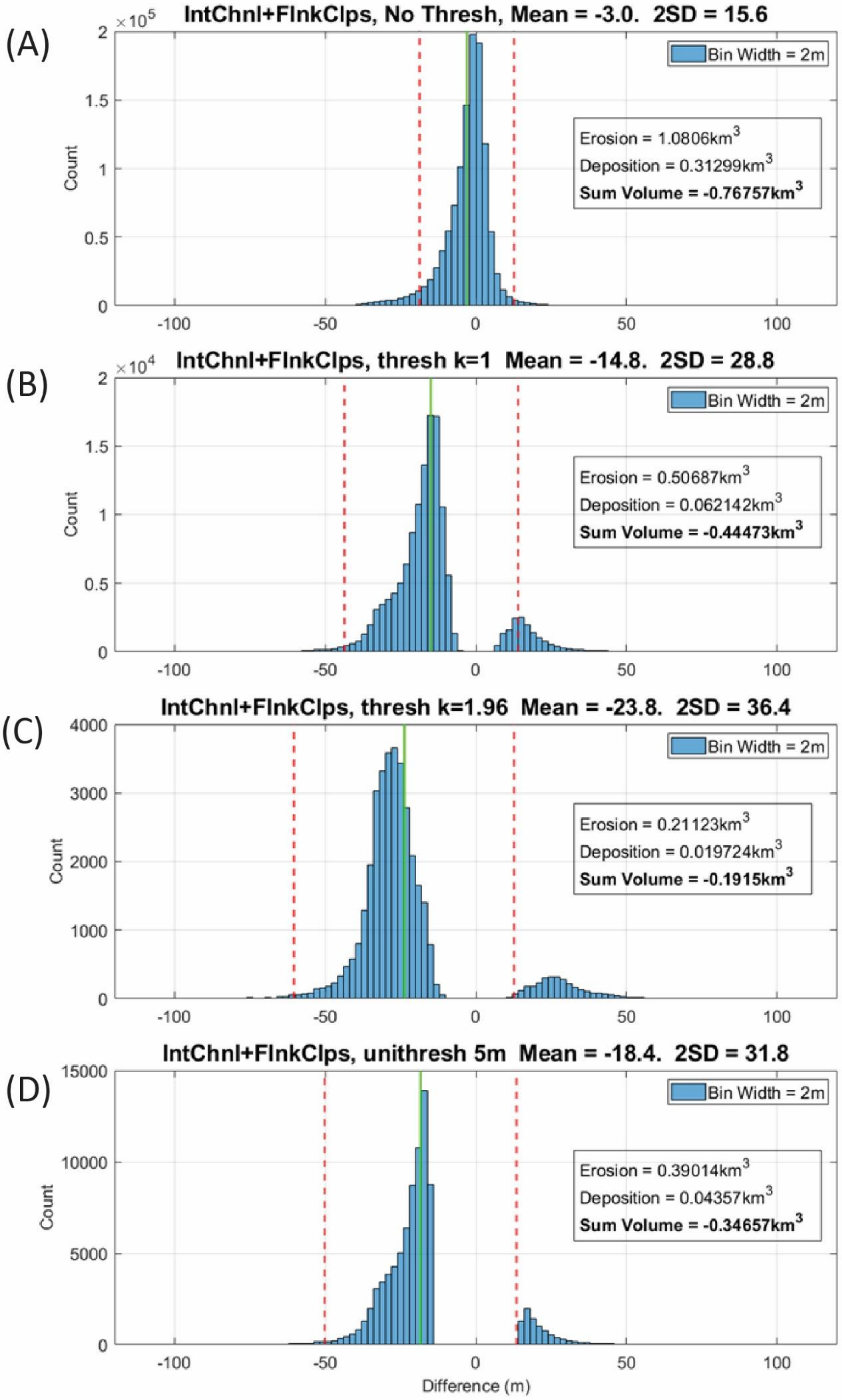
(A)
IntChnl+FlnkClps, No Thresh, Mean = -3.0. 2SD = 15.6
Bin Width = 2m
Erosion = 1.0806km^3
Deposition = 0.31299km^3
Sum Volume = -0.76757km^3
Count
(B)
IntChnl+FlnkClps, thresh k=1 Mean = -14.8. 2SD = 28.8
Bin Width = 2m
Erosion = 0.50687km^3
Deposition = 0.062142km^3
Sum Volume = -0.44473km^3
Count
(C)
IntChnl+FlnkClps, thresh k=1.96 Mean = -23.8. 2SD = 36.4
Bin Width = 2m
Erosion = 0.21123km^3
Deposition = 0.019724km^3
Sum Volume = -0.1915km^3
Count
(D)
IntChnl+FlnkClps, unithresh 5m Mean = -18.4. 2SD = 31.8
Bin Width = 2m
Erosion = 0.39014km^3
Deposition = 0.04357km^3
Sum Volume = -0.34657km^3
Count
Difference (m)

Supplementary Figure 10. Different methods for estimating uncertainties and limits of detection in seabed change along the deep-sea channel (international waters) (for location see Fig. 1). Plots show histograms of difference in seabed elevation change at individual grid cells along the axis of the Congo Canyon-channel system, between multibeam sonar surveys in September-October 2019 and October 2020. The modal elevation change (green line) and two standard deviations (red lines) are noted, along with the total volume of erosion in survey area (Supplementary Table 4). **(a)** All values of seabed change from every grid cell, without any limit of detection. **(b)** Grid cells removed if below a spatially varying limit of detection based on CUBE algorithm and k = 1 (raw CUBE-derived uncertainties). **(c)** Grid cells removed if below a spatially varying limit of detection based on the CUBE algorithm and k = 1.96 (i.e. 1.96 x raw CUBE-derived uncertainties) as in Mountjoy et al.[20]. **(d)** Grid cells removed if below a spatially fixed limit of detection set at 5 m. See Schimel et al.[69] for a detailed discussion of these different methods.
